# Deep medullary vein dysfunction is associated with cerebral microbleeds in cerebral small vessel disease

**DOI:** 10.3389/fnhum.2025.1636248

**Published:** 2025-08-29

**Authors:** Haiyuan Lan, Xinjun Lei, Zhihua Xu, Jie Yu, Huimei Wang

**Affiliations:** ^1^Department of Radiology, Lishui Hospital of Traditional Chinese Medicine Affiliated Zhejiang Chinese Medical University, Lishui, China; ^2^Department of Radiology, Tongde Hospital of Zhejiang Province, Hangzhou, China; ^3^Department of Neurology, Lishui People's Hospital, Lishui, China

**Keywords:** cerebral small vessel disease, deep medullary veins, cerebral microbleeds, extracellular fluid, susceptibility-weighted imaging

## Abstract

**Objective:**

This study investigated the association between deep medullary vein (DMV) dysfunction and the development of cerebral microbleeds (CMB) in patients with cerebral small vessel disease (CSVD), with a particular focus on the contribution of extracellular fluid accumulation.

**Methods:**

This was a cross sectional study. Clinical and imaging data from 176 patients with CSVD were consecutively collected between July 2024 and May 2025. DMV visibility was scored on a scale of 0–18 using susceptibility-weighted imaging (SWI). CMB were quantified on SWI magnitude images and categorized into three groups: absent CMB, mild CMB (1–2 lesions), and extensive CMB (≥3 lesions). Extracellular fluid volume was estimated using free water (FW) values derived from diffusion tensor imaging. Associations between DMV scores, FW values, and CMB burden were evaluated.

**Results:**

DMV scores were moderate positively correlated with both CMB burden and FW values (*r* = 0.460, *P* < 0.001; *r* = 0.549, *P* < 0.001, respectively), as well as between FW values and CMB burden (*r* = 0.561, *P* < 0.001). Patients in the extensive CMB group had significantly higher DMV scores and FW values compared to those in the absent CMB and mild CMB groups. Mediation analysis demonstrated that FW acted as a partial mediator in the relationship between DMV scores and CMB burden (β = 0.088, 95% CI: 0.048–0.152, *P* < 0.05). This mediating effect remained statistically significant after adjusting for age, sex, hypertension, diabetes, smoking, and hyperlipidemia (β = 0.054, 95% CI: 0.022–0.107, *P* < 0.05).

**Conclusion:**

DMV dysfunction is positively associated with CMB burden in CSVD, partially mediated by increased extracellular fluid accumulation. These findings suggest that impaired venous drainage and interstitial fluid retention may play a role in the pathogenesis of CMB.

## 1 Introduction

Cerebral microbleeds (CMB) are key neuroimaging markers of cerebral small vessel disease (CSVD), appearing as round or ovoid hypointense lesions (2–10 mm in diameter) on susceptibility-weighted imaging (SWI; Greenberg et al., 2009). Their presence and burden have been associated with various neurological outcomes, including cognitive impairment, ischemic stroke, and intracerebral hemorrhage (Yin et al., 2025; Lee et al., 2018; Wang et al., 2014). Despite their clinical significance, the pathophysiological mechanisms driving CMB formation remain incompletely understood. Previous investigations have predominantly focused on arterial etiologies, such as hypertensive vasculopathy and cerebral amyloid angiopathy, in explaining the development of CMB (Ungvari et al., 2017; Puy et al., 2024). In contrast, the contribution of venous abnormalities—particularly those involving the deep medullary veins (DMV)—has received limited attention.

Anatomically, DMV radiate from the periventricular white matter toward the subependymal veins, serving a critical role in venous drainage and cerebral homeostasis. Recent advances in SWI have facilitated more detailed visualization of venous structures, prompting increased research interest in DMV involvement in CSVD. Accumulating evidence suggests that DMV dysfunction is closely associated with the severity of CSVD (Tian et al., 2023; Xu et al., 2025). Proposed mechanisms include venous collagen deposition, resulting in venous hypertension and impaired drainage, particularly in distal territories. These changes may lead to increased extracellular fluid accumulation, contributing to microcirculatory disruption and CSVD progression. Moreover, excessive extracellular fluid may exacerbate blood-brain barrier dysfunction and impair the clearance of neurotoxic metabolites (Kern et al., 2024; Li et al., 2024), thereby promoting microstructural damage and potentially facilitating CMB formation (Hayden, 2024). On the basis of this emerging evidence, we hypothesized that DMV dysfunction may contribute to CMB pathogenesis, potentially mediated by pathological increases in extracellular fluid volume.

To test this hypothesis, the present study employed free water (FW) metrics derived from diffusion tensor imaging (DTI) to quantify extracellular fluid content and DMV visibility scores obtained from SWI to assess DMV function. The aim of this study was to examine the interrelationships between DMV scores, FW values, and CMB burden to elucidate potential venous-driven mechanisms underlying CMB development in CSVD.

## 2 Materials and methods

### 2.1 Study population

This study adopted a cross-sectional, prospective design, and was conducted at Lishui Hospital of Traditional Chinese Medicine affiliated with Zhejiang Chinese Medical University. A total of 176 patients with CSVD were consecutively enrolled in this study from July 2024 to May 2025. Inclusion criteria were as follows: (1) age >40 years; (2) availability of diagnostic-quality neuroimaging suitable for accurate evaluation of DMV; and (3) presence of cardiovascular comorbidities, including hypertension, dyslipidemia, smoking, or disorders of glucose metabolism. Exclusion criteria included: (1) intracranial pathologies such as tumors, hemorrhages, or acute ischemic lesions; (2) >70% stenosis of major cerebral arteries as detected by ultrasound; (3) secondary demyelinating diseases; (4) poor image quality precluding reliable DMV scoring; and (5) severe cardiac or renal dysfunction. The study protocol was approved by the Institutional Review Board of Lishui Hospital of Traditional Chinese Medicine affiliated Zhejiang Chinese Medical University (Ethics Committee Approval No. KY-2024058). Written informed consent was obtained from all participants prior to enrollment.

### 2.2 Imaging protocol and parameters

Neuroimaging was performed using a Magnetom Aera 1.5T MRI scanner (Siemens Healthcare, Erlangen, Germany) equipped with an eight-channel phased-array head coil. The imaging protocol included three core sequences: volumetric T1-weighted imaging (3D-T1WI), SWI, and DTI. The acquisition parameters were the following. (1) 3D-T1WI: repetition time (TR) = 2,000 ms; echo time (TE) = 2.8 ms; slice thickness = 1 mm; interslice gap = 0.5 mm; field of view (FOV) = 23 × 23 cm^2^; matrix = 256 × 256. (2) SWI: TR = 54 ms; TE = 40 ms; slice thickness = 2 mm; no interslice gap; FOV = 23 × 23 cm^2^; matrix = 256 × 256. (3) DTI: TR = 3,600 ms; TE = 85 ms; slice thickness = 5 mm; interslice gap = 1.5 mm; FOV = 23 × 23 cm^2^; matrix = 128 × 128; diffusion encoding directions = 30; b-values = 0, 1,000, and 2,000 s/mm^2^.

### 2.3 DMV scoring

**Figure 1:**
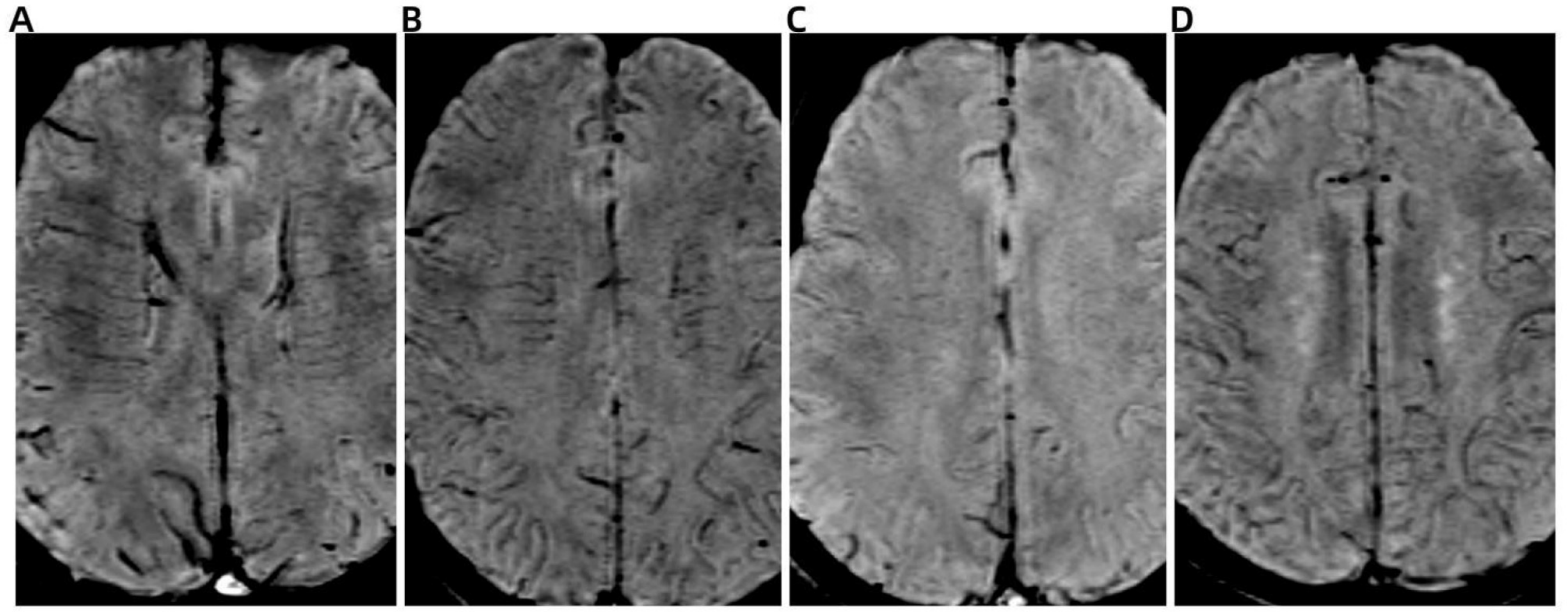
Representative images illustrating the deep medullary vein (DMV) scoring system on susceptibility-weighted imaging (SWI). **(A)** Score 0: DMV is continuous and clearly visible. **(B)** Score 1: DMV is partially visible with at least one discontinuity. **(C)** Score 2: DMV appears sparse, fragmented, or as thin, discontinuous hypointense streaks. **(D)** Score 3: DMV is not visible.

DMV score evaluation was conducted on SWI, focusing on six anatomically defined periventricular regions: the bilateral frontal, parietal, and occipital lobes adjacent to the lateral ventricles (Lee et al., 1996). DMV visibility in each region was graded according to established criteria (Chen et al., 2020) as follows: 0 points, DMV is continuous and clearly visible; 1 point, DMV is partially visible with at least one discontinuity. 2 points, DMV appear sparse, fragmented, or as thin, discontinuous hypointense lines. 3 points, there are no visible DMV. The total DMV score was calculated by summing the scores from all six regions, yielding a possible range from 0 to 18 (Figure 1). Higher scores indicated more severe venous disruption.

### 2.4 CMB assessment and group classification

CMB were identified on SWI as round or ovoid hypointense lesions with diameters ranging from 2 to 10 mm. A trained radiologist independently evaluated and quantified CMB using SWI magnitude images. Based on previously established criteria (Zhang et al., 2019; Heringa et al., 2014), patients were stratified into three groups according to their total CMB burden: no CMB: absence of CMB; Mild CMB: 1–2 CMB; Extensive CMB: ≥3 CMB.

### 2.5 Measurement of extracellular fluid

After visual quality control, raw diffusion data were first preprocessed using MRtrix3 Software, including noise suppression, correction of imaging artifacts, and rectification of echo planar imaging-induced geometric distortions. Quantification of extracellular FW was performed using the diffusion imaging in python (DIPY) software platform (https://dipy.org), employing a bi-compartmental FW elimination model (Hoy et al., 2014). Briefly, in each voxel, the signal was fitted to a two compartment model, including a FW compartment (isotropic tensor) and a tissue compartment (FW-corrected tensor). The estimated parameters were the fractional volume of the FW compartment. Subsequent co-registration aligned the 3D T1-weighted images with the *b* = 0 diffusion images. White matter FW values were extracted via tissue segmentation using the functional magnetic resonance imaging of the brain (FMRIB) Automated Segmentation Tool from the FMRIB Software Library (FSL) software suite, applied to the co-registered 3D-T1WI datasets. The FW metric ranged from 0 to 1, with higher values indicating greater extracellular FW content.

### 2.6 Statistical analysis

**Figure 2:**
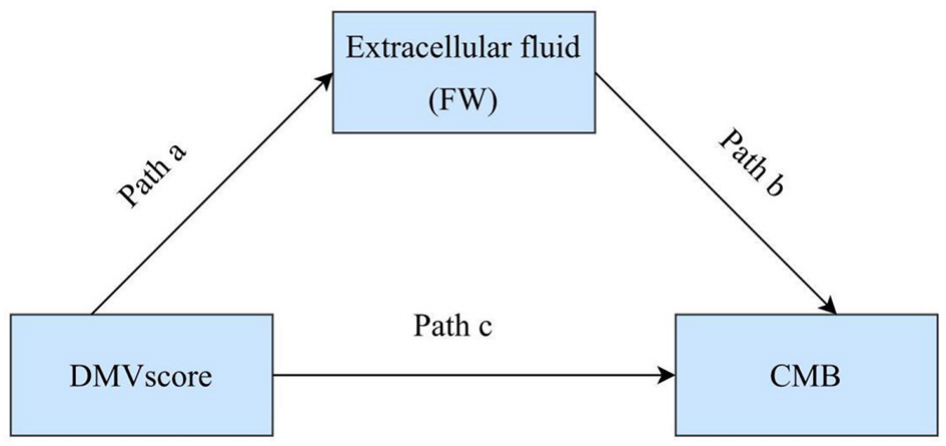
Schematic of the mediation model assessing the relationships between DMV score, free water (FW), and cerebral microbleeds (CMB).

Continuous variables with normal distributions were presented as mean ± standard deviation, while non-normally distributed data were reported as median and interquartile range (IQR). Categorical variables were expressed as frequencies and percentages (*n*, %). The association between DMV scores and CMB burden was evaluated using Spearman's rank correlation. Differences in DMV scores between CMB severity groups were assessed using the Kruskal-Wallis test. A multivariable general linear model was applied to identify factors independently associated with CMB burden. Mediation analysis was conducted to explore the interrelationships between DMV score, FW values, and CMB (see Figure 2 for schematic representation). All statistical analyses were performed using SPSS version 27.0 (IBM Corp., Armonk, NY, USA). A two-tailed *P*-value <0.05 was considered statistically significant.

## 3 Results

A total of 176 participants were included in the final analysis. The mean age was 62 ± 12 years, and 81 participants (46.0%) were male. Hypertension was present in 101 individuals (57.4%). CMB were detected in 69 participants (39.2%). The median DMV score was 4 (IQR: 1, 10), and the mean FW value was 0.25 ± 0.02 (Table 1).

**Table 1:** Baseline characteristics of the study population.

**Variables**	***n* = 176**
Age, years	62 ± 12
Sex, male	81 (46.0%)
Hypertension	101 (57.4%)
Diabetes	36 (20.5%)
Hyperlipidemia	53 (30.1%)
Smoking	39 (22.2%)
CMB	69 (39.2%)
Mild	46 (26.1%)
Extensive	23 (13.1%)
DMV	4 (1, 10)
FW	0.25 ± 0.02

CMB, cerebral microbleeds; DMV, deep medullary vein; FW, free water.

### 3.1 Inter-reader agreement for evaluation of deep medullary vein score

DMV scoring was independently performed by two neuroradiologists blinded to clinical and imaging data. The inter-reader intraclass correlation coefficients (ICCs) for the DMV score was 0.92.

### 3.2 Association between DMV scores, FW values, and CMB burden

**Figure 3:**
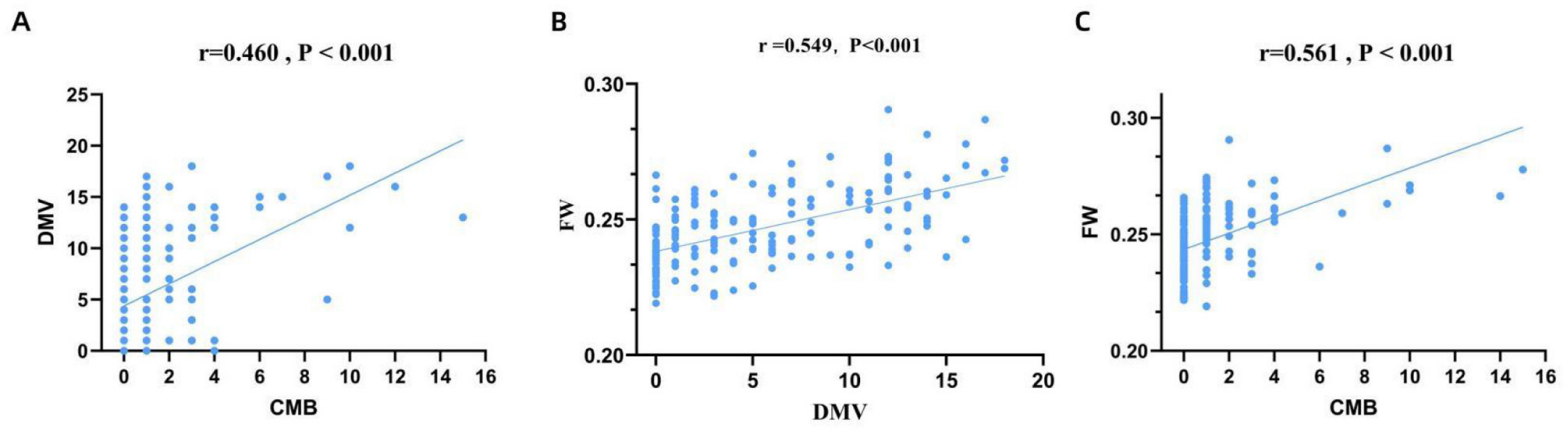
Correlation between DMV score, FW content and CMB burden. DMV, deep medullary vein; FW, free water; CMB, cerebral microbleeds.

**Figure 4:**
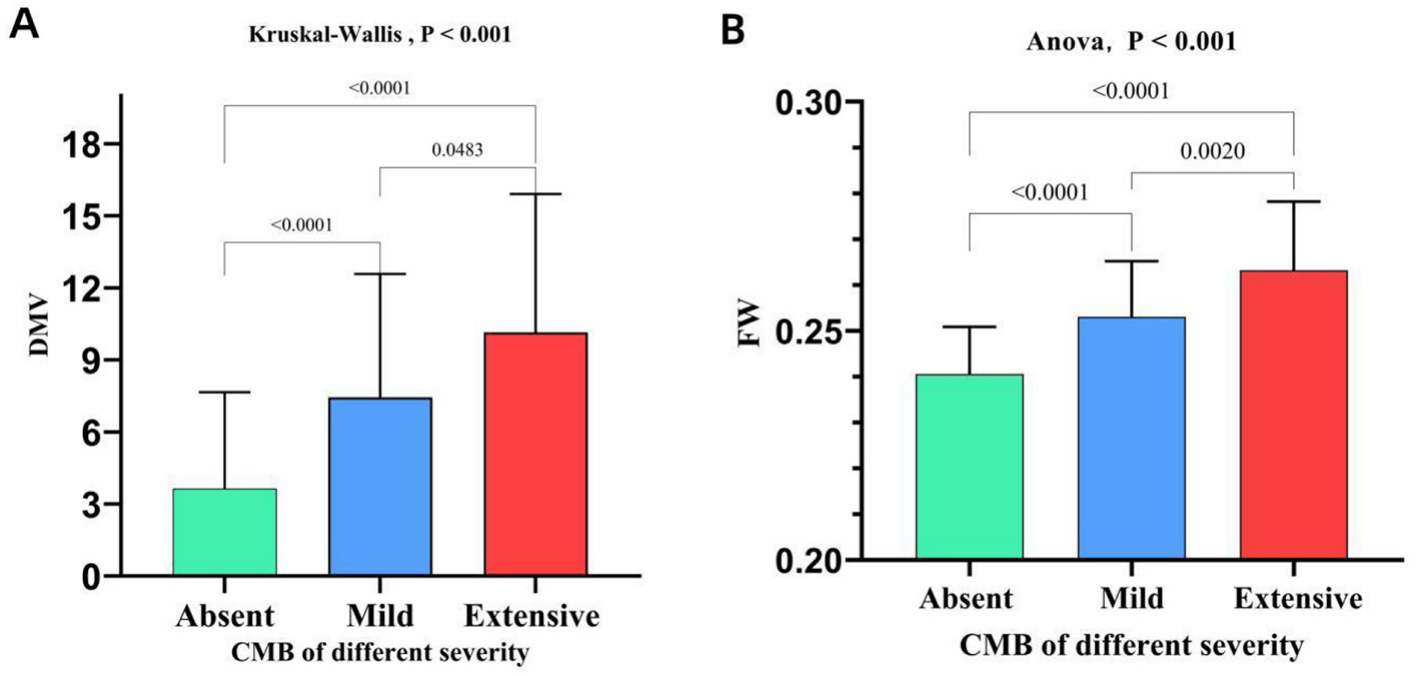
Comparison of DMV scores and FW content across groups stratified by CMB burden. DMV, deep medullary vein; CMB, cerebral microbleeds; FW, free water.

Spearman's rank correlation analysis demonstrated a significant moderate positive correlation between DMV scores and both CMB burden (*r* = 0.460, *P* < 0.001; Figure 3A) and FW values (*r* = 0.549, *P* < 0.001; Figure 3B), as well as between FW values and CMB burden (*r* = 0.561, *P* < 0.001; Figure 3C). The Kruskal-Wallis test or ANOVA test revealed significant differences in DMV scores and FW values across CMB severity groups (*P* < 0.001; Figures 4A, B). Pairwise comparisons showed that individuals with CMB had significantly higher DMV scores and FW values than those without CMB *(P* < 0.001). Furthermore, the extensive CMB group exhibited significantly higher DMV scores and FW values compared to the mild CMB group (*P* < 0.05).

### 3.3 Univariate and multivariate analysis of factors associated with CMB

Univariate general linear model analysis identified age, sex, and DMV scores as significantly associated with CMB burden (*P* < 0.05), while hypertension, hyperlipidemia, smoking status, and diabetes mellitus were not significantly associated (*P* > 0.05; Table 2). In multivariate analysis, DMV score and sex emerged as independent risk factors for CMB (*P* < 0.05; Table 3).

**Table 2:** Univariate analysis of factors associated with cerebral microbleeds (CMB).

**Variables**	**β (95% CI)**	***P*-value**
Age, years	0.066 (0.031, 0.100)	<0.001
Sex, male	1.252 (0.414, 2.090)	0.004
Hypertension	0.516 (−0.346, 1.378)	0.239
Diabetes	0.326 (−0.734, 1.386)	0.544
Hyperlipidemia	−0.394 (−1.337, 0.584)	0.410
Smoking	−0.894 (−1.915, 0.128)	0.086
DMV	0.231 (0.156, 0.306)	<0.001

DMV, deep medullary vein.

**Table 3:** Multivariate analysis of independent predictors of cerebral microbleeds (CMB).

**Variables**	**β (95% CI)**	***P*-value**
Age, years	0.010 (−0.028, 0.049)	0.605
Sex, male	1.188 (0.425,1.951)	0.002
DMV	0.215 (0.125, 0.304)	<0.001

DMV, deep medullary vein.

### 3.4 Mediation analysis of DMV scores, FW values, and CMB

The mediation analysis in Model 1 demonstrated a significant direct effect of DMV on CMB (β = 0.143, 95% CI: 0.058–0.228, *P* < 0.05), with extracellular FW partially mediating the relationship between DMV scores and CMB burden (β = 0.088, 95% CI: 0.048–0.152, *P* < 0.05). In Model 2, after adjusting for age, sex, hypertension, diabetes, smoking, and hyperlipidemia, the direct effect (β = 0.158, 95%CI: 0.067–0.250, *P* < 0.05) and indirect effect (β = 0.054, 95%CI: 0.022–0.107, *P* < 0.05) remained statistically significant (Table 4).

**Table 4:** Mediation analysis results examining the relationships between DMV score, FW, and CMB.

**Model**	**Path a**	**Path b**	**Path c-direct effect**	**Path ab-indirect effect**
	**(**β**, 95%CI)**	**(**β**, 95%CI)**	**(**β**, 95%CI)**	**(**β**, 95%CI)**
Model 1	0.102 (0.078, 0.127)^*^	0.863 (0.420, 1.306)^*^	0.143 (0.058, 0.228)^*^	0.088 (0.048, 0.152)^*^
Model 2	0.060 (0.032, 0.089)^*^	0.900 (0.430, 1.368)^*^	0.158 (0.067, 0.250)^*^	0.054 (0.022, 0.107)^*^

^*^P < 0.05.

Model 1: DMV score serving as predictor, FW as mediator, and CMB as outcome; Model 2: Model 1, adjusted for age, sex, hypertension, diabetes, smoking, and hyperlipidemia.

DMV, deep medullary vein; FW, free water; CMB, cerebral microbleeds.

## 4 Discussion

This study demonstrated that patients with CMB exhibited significantly higher DMV scores compared to those with mild or absent CMB. Furthermore, DMV scores were independently associated with CMB burden, and this relationship was partially mediated by extracellular FW. The mediating effect of FW remained statistically significant after adjustment for age, hypertension, sex, diabetes, smoking, and hyperlipidemia, suggesting that DMV dysfunction may contribute to CMB pathogenesis through mechanisms involving extracellular fluid accumulation.

As a key component of the cerebral venous drainage system, the DMV plays a critical role in maintaining cerebral hemodynamic balance. Histopathologically, DMV walls consist solely of a single layer of endothelial cells and delicate connective tissue, lacking smooth muscle and elastic fibers (Lahna et al., 2022). Moreover, the DMV has small diameters and long courses with slow blood flow, making them prone to stenosis due to venous collagen deposition. Additionally, the DMV has few anastomotic branches, resulting in limited compensatory capacity once stenosis or occlusion occurs. This anatomical configuration confers limited autoregulatory capacity, making the DMV particularly vulnerable to hemodynamic disturbances. Such vulnerability may predispose the DMV to dysfunction, which can disrupt the cerebral microenvironment.

Progressive collagen deposition in the DMV, often driven by aging and cardiometabolic conditions such as hypertension, dyslipidemia, and diabetes, leads to venous narrowing and luminal stenosis (Moody et al., 1995). Chronic stenosis elevates venous pressure and promotes blood-brain barrier disruption (Keith et al., 2017)—a pathological feature closely linked to CMB formation in preclinical studies (Fisher et al., 2010). Elevated venous pressure also increases vascular permeability, resulting in extravasation of fluid and a rise in extracellular fluid volume. Impaired venous outflow from distal microvasculature may lead to interstitial edema in the cerebral white matter, further contributing to extracellular fluid accumulation (Lan et al., 2024). Excess extracellular fluid impairs the clearance of β-amyloid, facilitating its pathological accumulation. Deposited β-amyloid compromises arterial smooth muscle integrity, enabling the leakage of intravascular components—such as erythrocytes, plasma, and hemoglobin—into the interstitial space (Stokum et al., 2021). The breakdown of these components results in hemosiderin deposition, which appears as hypointense signals on SWI, characteristic of CMB. Additionally, extracellular fluid accumulation can damage axonal and myelin structures, eliciting neuroinflammatory responses that further contribute to CMB development (Sumbria et al., 2016). Neuroinflammation exacerbates blood-brain barrier dysfunction and promotes microvascular hyperpermeability and erythrocyte extravasation (Wang et al., 2021), thereby establishing a vicious cycle. Importantly, neuroinflammation itself may elevate extracellular fluid levels, reinforcing this pathogenic feedback loop.

In this study, DMV scores demonstrated a moderate positive correlation with CMB burden (*r* = 0.460). On one hand, this suggests complex pathological mechanisms underlying CMB, where age, sex, and hypertension are established high-risk factors for its onset and progression of CMB (Ungvari et al., 2017; Elmståhl et al., 2019; Fandler-Höfler et al., 2024). On the other hand, it indicates interindividual variation in both the degree of DMV stenosis and compensatory capacity. So, in the present study, the mediating role of extracellular FW in the relationship between DMV scores and CMB remained statistically significant even after adjusting for age, sex, vascular risk factors (hypertension, diabetes, smoking, and hyperlipidemia). This finding not only reinforces the robustness of our results but also substantiates a mechanistic link between DMV dysfunction, increased FW content, and CMB burden.

Moreover, our study revealed a significant direct effect between DMV scores and CMB. These findings not only confirm the pivotal role of DMV dysfunction in the pathogenesis of CMB, but also suggest the potential involvement of other unmeasured mediating pathways (e.g., blood-brain barrier disruption or neuroinflammation) in CMB development.

This study has several limitations. First, it was conducted at a single center with a relatively modest sample size, which may introduce selection bias. Future research should incorporate multi-center cohorts and larger sample sizes to enhance generalizability. Second, the cross-sectional design limits the ability to assess temporal relationships between DMV impairment, FW elevation, and CMB development. Longitudinal studies are needed to elucidate causality. Third, clinical cognitive data were not collected, preventing direct evaluation of whether elevated FW contributes to cognitive decline in individuals with CMB. Fourth, factors potentially contributing to elevated FW values may include blood-brain barrier dysfunction and neuroinflammation. However, the absence of blood-brain barrier function and neuroinflammation data in our study precluded their inclusion as confounding factors for statistical adjustment.

In conclusion, the dysfunction of the DMV partially mediates the formation of CMB in patients with CSVD through increased extracellular FW. These findings suggest that impaired venous drainage and interstitial fluid accumulation may constitute key pathological processes in the development of CMB.

## Data Availability

The original contributions presented in the study are included in the article/supplementary material, further inquiries can be directed to the corresponding author.
